# COVID-19 vaccine acceptance and associated factors among people living with HIV in the Middle East and North Africa region

**DOI:** 10.4102/sajhivmed.v23i1.1391

**Published:** 2022-08-24

**Authors:** Rahma Mohamed, Trenton M. White, Jeffrey V. Lazarus, Amany Salem, Reham Kaki, Wafa Marrakchi, Sara G. M Kheir, Ibrahim Amer, Fida M Ahmed, Maie A Khayat, Nabeela Al-Abdullah, Batool Ali, Roaa Sultan, Bandar Alamri, Anouf Abdulmajid, Ikbal Kooli, Mohamed Chakroun, Tariq A. Madani, Gamal Esmat, Ahmed Cordie

**Affiliations:** 1 Endemic Medicine Department, Kasr Alaini School of Medicine, Cairo University Hospitals, Cairo, Egypt; 2 Kasr Al-Aini HIV and Viral Hepatitis Fighting Group, Kasr Alaini School of Medicine, Cairo University Hospitals, Cairo, Egypt; 3 Barcelona Institute for Global Health (ISGlobal), Hospital Clinic, University of Barcelona, Barcelona, Spain; 4 Faculty of Medicine and Health Sciences, University of Barcelona, Barcelona, Spain; 5 Department of Public Health, Kasr Alaini School of Medicine, Cairo University, Cairo, Egypt; 6 Department of Infectious Disease, Infection Control and Environmental Health, Faculty of Medicine, King Abdulaziz University, Jeddah, Saudi Arabia; 7 Infectious Diseases Department, Faculty of Medicine, University Hospital of Monastir, Monastir, Tunisia; 8 Disease Control Directorate, Federal Ministry of Health, Khartoum, Sudan; 9 Department of Hepatology, Gastroenterology and Infectious Diseases, Faculty of Medicine, Kafr El-Sheikh University, Kafr El-Sheikh, Egypt; 10 College of Nursing, King Abdulaziz University, Jeddah, Saudi Arabia; 11 Department of Infectious Diseases, East Jeddah General Hospital, Jeddah, Saudi Arabia; 12 Infectious Diseases Department, Armed Forces College of Medicine, Cairo, Egypt

**Keywords:** COVID-19, COVID-19 vaccine, vaccine acceptance, Middle East, HIV

## Abstract

**Background:**

Identifying coronavirus disease 2019 (COVID-19) vaccine acceptance and associated factors among people living with HIV (PLHIV) in the Middle East and North Africa region is important to meet the need for broad-scale vaccination against COVID-19.

**Objectives:**

To investigate the COVID-19 vaccine acceptance rate and factors among PLHIV in the Middle East and North Africa region.

**Method:**

An online cross-sectional survey was conducted among PLHIV currently living in Egypt, Tunisia and Saudi Arabia between March 2021 and August 2021.

**Results:**

Of the 540 respondents, 19.3% reported already being vaccinated against COVID-19 (*n* = 104), 32.0% responded ‘definitely yes’ (*n* = 173), and 13.3% responded ‘probably yes’ (*n* = 72) for intention to receive a COVID-19 vaccine, with an overall COVID-19 vaccine acceptance rate of 64.6% among PLHIV in the region. The most significant predictors of COVID-19 vaccine acceptance included feeling less worried about COVID-19 transmission post-vaccination (221.0% higher odds), and believing the disease is vaccine-preventable (160.0% higher odds). Reported barriers to COVID-19 vaccine acceptance include concerns about vaccine effectiveness and belief that HIV medications protect against COVID-19 transmission, living in a rural area and reporting less-frequent engagement with HIV care. Nine out of 10 participants reported that the chances of them getting COVID-19 vaccine would increase if given adequate information and if their doctor recommended it.

**Conclusion:**

Findings of the study can help researchers, health officials, and other health system actors understand the predictors and barriers to COVID-19 vaccine acceptance reported by PLHIV. This understanding could inform the future planning of interventions tailored to PLHIV.

## Introduction

For four decades, HIV/AIDS has been one of the world’s most serious public health challenges, with an estimated 36 million AIDS-related deaths worldwide since the start of the pandemic, and nearly 38 million people currently living with HIV.^1^ Despite having the lowest estimated HIV prevalence rates in the world (< 0.1%), the Middle East and North Africa (MENA) region is an area of growing concern, with a 47% increase in new infections and a 57% increase in AIDS-related deaths compared to 2010.^2,3^ In addition, in this region it is estimated that only 52% of people living with HIV (PLHIV) are aware of their status, and only 43% of all PLHIV are on antiretroviral treatment.^2,4^

In the midst of global and regional efforts to control the HIV epidemic, the novel coronavirus disease 2019 (COVID-19) pandemic emerged, continuing to threaten hard-won gains made against HIV through service disruption, COVID-19 myths among PLHIV, and potential unknown implications of Long-COVID and HIV comorbidity.^5,6,7^ As of December 2021, more than 17 million cases of COVID-19 and some 312 000 deaths have been reported across the region.^8^

Several MENA countries adopted strict containment measures to decrease the spread of COVID-19, including closing borders, schools, religious sites and public places, and imposing a full lockdown for certain periods of time.^9^ However, some of these measures intensified existing challenges to HIV care by impeding access to prevention, clinical care and treatment services,^5,6^ and by generating or exacerbating stress, depression and isolation among PLHIV.^10^

Increased risk of COVID-19 mortality among PLHIV, compared to their HIV-negative counterparts, was demonstrated in South Africa and the United Kingdom.^11,12,13^ Similarly, a study of over 15 000 cases of COVID-19 in PLHIV from 24 countries conducted by the World Health Organization found that PLHIV were at higher risk of severe or critical illness at the time of hospital admission and in-hospital mortality after controlling for age, gender and comorbidity burden.^14^

Vaccination is the most effective intervention to prevent severe illness and death from COVID-19.^15^ As of December 2021, over eight billion doses of COVID-19 vaccines have been administered globally, and 56% of the world population has received at least one dose.^16^ Despite this global progress, vaccination coverage remains very low in low- and lower-middle income countries, including those in the MENA region.^17^

Slow COVID-19 vaccine coverage reflects global supply issues as well as decreased demand due to vaccine hesitancy. Vaccine hesitancy, defined as ‘a delay in acceptance or refusal of vaccination despite availability of vaccination services’, has been a growing concern worldwide,^18^ prompting the World Health Organization to name it one of the top 10 global health threats in 2019. This challenge has grown during the COVID-19 pandemic.^19^ Existing studies on public perceptions and acceptance of COVID-19 vaccines in the MENA region illustrate that the Middle East is among the regions with the lowest rates of vaccine acceptance globally.^20,21^ Vaccine hesitancy could jeopardise the success of COVID-19 vaccination programmes and undermine efforts to ensure high vaccination coverage rates, especially among vulnerable populations, such as PLHIV. The objectives of this study were to evaluate the acceptance of the COVID-19 vaccine and influencing factors among PLHIV in the MENA region.

## Methods

### Study design and sample

The study is a cross-sectional web-based anonymous survey, designed and administrated using Google Forms. People living with HIV aged 18 years or older, whether male, female or transgender, and living in Egypt, Tunisia or the Kingdom of Saudi Arabia between March 2021 and August 2021 were included. Those who disagreed to give consent before submitting their responses were excluded. A sample of PLHIV was calculated using purposive quota non-probability methods. The research team then contacted and enrolled PLHIV who were on follow-up in their HIV clinics via telephone and email. In addition, there are many community-based organisations providing services to PLHIV in each country that facilitated distribution of the survey to their networks through emails and social media platforms (Facebook and WhatsApp). Participants were informed that their participation was voluntary, given a brief introduction to the study and its objective, and requested to provide informed consent prior to accessing the questionnaire.

Sample size was calculated to determine the minimum proper sample size for the prevalence of acceptance of COVID-19 vaccine among PLHIV in the MENA region. Reviewing the literature revealed that no previous studies had been performed on PLHIV in the MENA region; however, the rate of acceptance of COVID-19 vaccine among the general population ranged from 23.6% to 77.6%,^19,21^ with an average of 50.6%. If we assumed that this was the true population prevalence, we needed to study 164 participants to be able to achieve 80.0% power, setting the alpha error at 0.05 and the prevalence error margin at 5.0% using the generic Z test. Sample size calculation was done by StatCalc, Epi Info version 7 for MS Windows (Centers for Disease Control and Prevention, Atlanta, Georgia, United States [US]).

### Data collection tool

The closed-ended questionnaire addressed: (1) sociodemographic characteristics of participants; (2) individual health, including HIV-related health; (3) COVID-19 vaccination and intention; and (4) a health belief model (HBM) of COVID-19 disease and vaccination (see Online Appendix 2 File 1). The questionnaire was developed in English and forward translated to Arabic by a professional interpreter. The forward translations were then back translated by bilingual members of the research team who, through majority consensus, made decisions about the semantic, idiomatic, experiential and conceptual equivalence of the translated items. Before distribution, the questionnaire’s content validity was tested and verified by local HIV experts and through pilot testing among fewer than 10 PLHIV, where the questionnaire was estimated to take about 15 min to complete. The data were collected anonymously and assigned a unique identification in the phase of data entry.

### Sociodemographic and health-related variables

Participants were asked to report their age, gender, marital status, educational attainment, employment status, country and area of residence (rural or urban). Participants were also asked if they had other existing chronic diseases (e.g. diabetes, hypertension, lung disease, liver disease, kidney disease, heart disease, and/or malignancy), and to rate their overall health status. Participants reported prior receipt of vaccination against seasonal influenza this year and if they were previously COVID-19 tested or hospitalised because of COVID-19. Participants were also asked if they were currently receiving their HIV medications, and for their most recent CD4 count and HIV viral load.

### COVID-19 vaccination and intention

Participants reported if they had already been vaccinated against COVID-19, and, if the answer was ‘no’, they were asked the follow-up question, ‘When a COVID-19 vaccine becomes available to you, will you take it?’, to assess their intention to accept a COVID-19 vaccine on a five-point scale (‘definitely no’ to ‘definitely yes’). Participants were coded by the authors as the vaccine-accepting group if they answered ‘yes’ to already being vaccinated, or ‘definitely yes’ or ‘probably yes’ to the intention question. The vaccine non-accepting group included those who answered, ‘probably no’, ‘not sure’ or ‘definitely no’ to the intention question. All participants were also asked if they ‘would pay a fee to be vaccinated, if needed’, on a five-point scale (‘definitely no’ to ‘definitely yes’).

### Health belief model of COVID-19 disease and vaccination

The questionnaire items on participants’ beliefs about COVID-19 vaccination were partially derived from the HBM. This model is used to describe individuals’ health-related behaviour according to their perception of predisposition, efficacy and outcomes.^22^ Questions included main domains that affect health behaviour: perceived susceptibility to COVID-19 infection and severity (three items), perceived benefits of a COVID-19 vaccine (two items), perceived vaccination barriers (seven items), and cues to action (two items). Each item was based on a five-point Likert scale (‘strongly agree’, ‘agree’, ‘neither disagree or agree’, ‘disagree’ or ‘strongly disagree’). Chronbach’s alpha tested the internal reliability of the 14-item scale.

### Statistical analysis

Multivariable ordered logistic regression analyses were used to report the odds ratios across the sample for vaccine acceptance with: (1) a sociodemographic model; (2) an HIV-related and overall individual health model; and (3) an HBM model. A likelihood ratio test was used to test the models’ fit by nesting the sociodemographic model within the other two models.

### Ethical considerations

This study was approved by the University of Kafr El-Sheikh Research Ethics Committee. Approval code: MKSU 13-3-20. Also, the Institutional Review Boards of Jeddah (No.H-02-J002/2021) approved this study.

## Results

### Sociodemographic and individual health data

Descriptive characteristics of the respondents are provided in Table 1 and Table 2. Overall, 540 respondents completed the survey, 72% of whom were male (*n* = 389), and 54.8% aged 18–39 years (*n* = 296). Forty-two per cent (42%) of respondents (*n* = 227) were from Egypt, 34.4% were from Saudi Arabia (*n* = 186), and 23.5% were from Tunisia (*n* = 127), with a higher proportion of participants (85.2%) living in urban areas (*n* = 460). Responses to the HBM are provided in Online Appendix 1, Table 1.

**TABLE 1:** Sociodemographic characteristics of the sample (*n* = 540).

Variables	*n*	%
**Age**		
18–29	103	19.1
30–39	193	35.7
40–49	126	23.3
50–59	81	15.0
60–69	34	6.3
70–79	3	0.6
**Gender**		
Male	389	72.0
Female	148	27.4
Don’t wish to disclose	3	0.6
**Country**		
Egypt	227	42.0
Saudi Arabia	186	34.4
Tunisia	127	23.5
**Education**		
Primary	89	16.5
Preparatory	73	13.5
Secondary	134	24.8
Technical	39	7.2
University and above	205	38.0
**Marital status**		
Single	209	38.7
Married	229	42.4
Divorced	57	10.6
Widow	45	8.3
**Employment status**		
Employed	267	49.4
Retired	37	6.9
Student	26	4.8
Unemployed	210	38.9
**Area of residence**		
Rural	80	14.8
Urban	460	85.2

**TABLE 2:** HIV-related and overall health of individual participants (*n* = 540).

Variable	*n*	%
**HIV-infection specific questions**		
On HIV treatment		
Yes	524	97.0
No	16	16.0
Last time checked CD4 count		
6 months ago	300	55.6
A year ago	53	9.8
More than one year	187	34.6
Last CD4 count		
< 250	40	7.4
250–500	102	18.9
> 500	151	28.0
I don’t know	247	45.7
Last time checked viral load		
6 months ago	343	63.5
A year ago	59	10.9
More than one year	138	25.6
Undetectable viral load		
Yes	337	62.4
No	73	13.5
Don’t know	130	24.1
**General health-related questions**		
Other chronic conditions		
Yes	140	25.9
No	400	74.1
How do you rate your overall health?		
Very good	267	49.4
Good	170	31.5
Fair	75	13.9
Poor	21	3.9
Very poor	7	1.3
Vaccinated against influenza		
Yes	122	22.6
No	418	77.4
**COVID-19 experience**		
Tested for COVID-19		
Yes	210	38.9
No	330	61.1
Test results		
Negative	155	73.8
Positive	55	26.2
Hospitalised		
Yes	11	20.0
No	44	80.0

COVID-19, coronavirus disease 2019

### Overall COVID-19 vaccine acceptance

**FIGURE 1:**
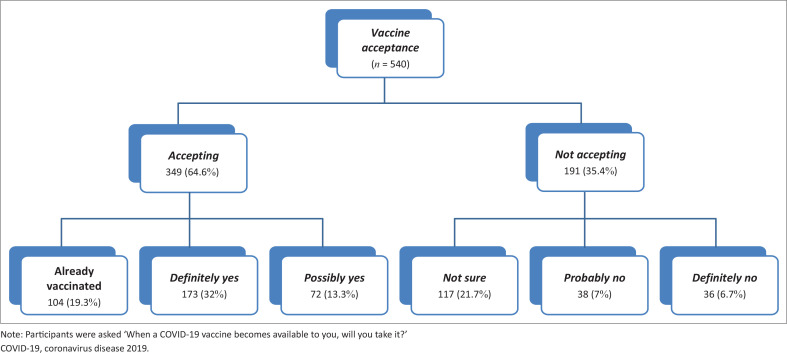
COVID-19 vaccine acceptance among Middle East and North Africa people living with HIV (*n* = 540).

**FIGURE 2:**
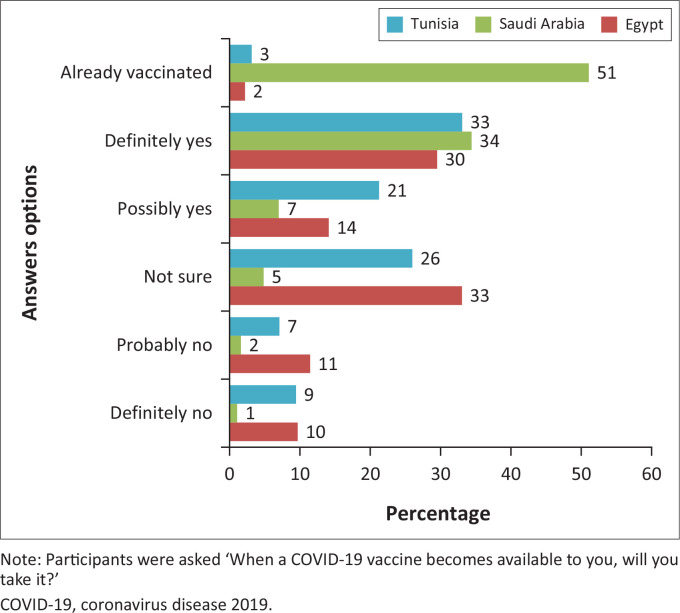
COVID-19 vaccination intention by country (Egypt [*n* = 227], Saudi Arabia [*n* = 186], Tunisia [*n* = 127]).

**FIGURE 3:**
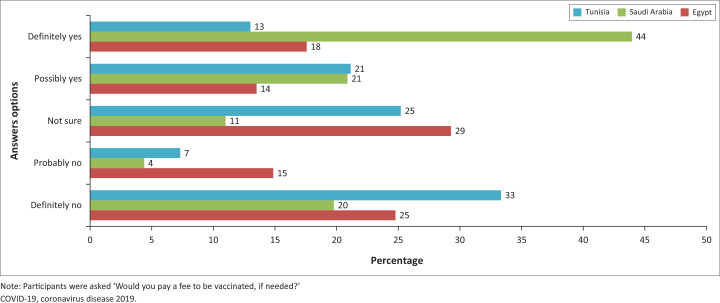
Willingness to pay for a COVID-19 vaccine by country.

Figure 1 and Figure 2 demonstrate COVID-19 vaccine acceptance among PLHIV in the studied countries. Overall, 64.6% of the sample reported acceptance. Of these, 19.3% (*n* = 104) had already been vaccinated against COVID-19, 32.0% responded ‘definitely yes’ (*n* = 173), and 13.3% responded ‘probably yes’ (*n* = 72) for intention to receive a COVID-19 vaccine, with relative variation in acceptance rate between the three countries. Only 6.7% responded ‘definitely no’ (*n* = 36), 7.0% reported ‘probably no’ (*n* = 38), and 21.7% ‘not sure’ (*n* = 117). A higher proportion of participants from Saudi Arabia (65%) were willing to pay a fee than the average (39.0%) to get the vaccine (Figure 3).

### Factors associated with COVID-19 vaccine acceptance

In the three multivariate models assessing vaccine acceptance, the HBM demonstrated the strongest fit with a Pseudo R2 = 0.30 (*P* = 0.00), compared with the individual health model (Pseudo R2 = 0.15; *P* = 0.00) and sociodemographic model (Pseudo R2 = 0.03; *P* = 0.00). Cronbach’s alpha test for the HBM was estimated to be 0.83. In the nested likelihood ratio tests, both models demonstrated a statistically significant improvement in model fit by including the sociodemographic variables (*P* = 0.00) (Table 3).

**TABLE 3:** Factors for vaccine acceptance among (a) sociodemographic characteristics, (b) HIV-related and overall health, and (c) COVID-19 HBM perceptions.

Questions	Odds ratio	95% CI	*P*	*N*	Likelihood ratio coefficient for nested models
**(a) Sociodemographic characteristics**	-	-	-	537	-
Education (with university degree vs without)	1.24	0.84–1.85	0.280	-	-
Employed versus non-employed	1.20	0.82–1.75	0.350	-	-
Age	1.16	0.99–1.38	0.073	-	-
Gender	1.00	0.65–1.52	0.993	-	-
Married versus non-married	0.97	0.66–1.43	0.896	-	-
Rural versus urban	0.44	0.26–0.70	0.001	-	-
**(b) HIV-related and overall health**	-	-	0.000	395	251.26
Currently receiving ART versus not	4.35	0.85–22.31	0.078	-	-
Overall health (very good and good vs fair, poor and very poor)	1.07	0.78–1.46	0.686	-	-
Last viral load check more than versus less than one year	0.87	0.60–1.27	0.480	-	-
Most recent viral load undetectable versus not	0.83	0.43–1.60	0.577	-	-
Presence of other chronic morbidity versus not	0.55	0.30–1.00	0.050	-	-
Last CD4+ count check more than versus less than one year	0.50	0.35–0.70	0.000	-	-
(c) COVID-19 health belief model statements*	-	-	0.000	433	260.95
Vaccination is a good idea because I will feel less worried about getting COVID-19.	2.21	1.60–3.06	0.000	-	-
COVID-19 can be prevented by vaccination.	1.60	1.16–2.21	0.004	-	-
The chances of me getting vaccinated against COVID-19 will increase if my doctor recommends me.	1.42	1.02–1.98	0.041	-	-
I am concerned that the COVID-19 vaccine is not effective as I have a weak immune system or CD4+ < 200.	1.07	0.73–1.58	0.720	-	-
I have concerns about the cost of getting the COVID-19 vaccine.	1.05	0.79–1.38	0.753	-	-
I am worried about the possible drug-drug interaction with my HIV medications.	1.04	0.75–1.43	0.817	-	-
I am concerned that COVID-19 vaccine is not recommended for me as I have a weak immune system or CD4+ < 200.	1.02	0.69–1.50	0.924	-	-
COVID-19 is a dangerous health threat especially to me as a patient with chronic disease.	1.01	0.72–1.42	0.961	-	-
If I get COVID-19, I will be very sick and probably need hospitalisation.	0.96	0.70–1.32	0.821	-	-
My chances of getting COVID-19 in the next few months is great	0.80	0.58–1.10	0.174	-	-
I am worried about the possible side effects of COVID-19 vaccine.	0.79	0.58–1.07	0.126	-	-
The chances of me getting vaccinated against COVID-19 will increase if I was given adequate information about it	0.75	0.56–1.00	0.054	-	-
I’ve heard my HIV medicines protect me from getting COVID-19, so I do not need the vaccine.	0.65	0.51–0.84	0.001	-	-
I have concern about the effectiveness of the COVID-19 vaccine.	0.52	0.38–0.72	0.000	-	-

Note: Statement response options ‘strongly agree’ and ‘agree disagree’ were combined and compared to response options ‘neither disagree or agree’, ‘disagree’, and ‘strongly disagree’.

ART, antiretroviral treatment; CI, confidence interval; COVID-19, coronavirus disease 2019.

In the sociodemographic model, living in a rural area is associated with 58% lower odds of accepting a COVID-19 vaccine compared to living in an urban area, whereas the other variables hold no significant association.

In the model assessing individual HIV-related and overall health of participants, those reporting their most recent CD4+ cell check being more than one year ago demonstrated 50% lower odds of COVID-19 vaccine acceptance compared to those reporting a recent check within one year, after controlling for sociodemographic characteristics.

In the COVID-19 HBM after controlling for sociodemographic characteristics, feeling less worried about getting COVID-19 post-vaccination (221% higher odds), believing COVID-19 is vaccine-preventable (160% higher odds), and a doctor recommendation (142% higher odds) were all associated with higher acceptance. Conversely, believing HIV medication protects against COVID-19 transmission (35% lower odds) and concerns about vaccine effectiveness (48% lower odds) were associated with lower acceptance. Other evaluated items held no significant associations.

## Discussion

Our study investigated COVID-19 vaccine acceptance among PLHIV in Egypt, Tunisia and Saudi Arabia, including associated factors such as sociodemographic characteristics, individuals’ HIV-related health, vaccine perceptions and health beliefs. The overall COVID-19 vaccine acceptance rate among PLHIV who participated in this survey is 64.6%, with wide variation between countries (92.0% in Saudi Arabia, 57.0% in Tunisia, and 46.0% in Egypt). These regional differences in vaccine acceptance are reflected by vaccination coverage in Saudi Arabia, where 71.2% of the general population have received at least one dose of a COVID-19 vaccine, compared with Tunisia (52.9%) and Egypt (27.3%), as of 12 December 2021.^23^ A previous study conducted in January 2021 in France showed that 71.3% of PLHIV would accept COVID-19 vaccines, while 28.7% demonstrated hesitancy.^24^ In eight cities in China between January and February 2021, 57.2% of PLHIV reported willingness to be vaccinated against COVID-19.^25^ Compared to studies conducted in the MENA region, the acceptance of a COVID-19 vaccine among our study participants was higher than the general population in Jordan (28.4%), Egypt (43.0%), Oman (57.0%), Lebanon (58.8%), and Qatar (60.0%); similar to that in Saudi Arabia (64.7%); and lower than in Kuwait (67.0%), Somalia (76.8%) and Iraq (77.6%).^20,26,27,28,29,30,31,32,33^

Our study is consistent with others regarding weaker COVID-19 vaccine acceptance among those residing in rural areas^34^ and regarding age and gender among French PLHIV.^24^ Compared to the general population in MENA region, there was mixed evidence with respect to the relationship between age and COVID-19 vaccine acceptance. Some studies demonstrate higher acceptance of COVID-19 vaccines among younger age groups,^30,32,35^ and others report higher acceptance among older age groups.^29,31^ Our results demonstrate comparable vaccine acceptance across gender, marital status and education level, differing from studies that demonstrate higher acceptance among male versus female participants, married versus non-married, and higher versus lower education level.^29,30,31,32,35^ The low acceptance rate among rural communities may reflect socio-economic disadvantage, lack of knowledge regarding COVID-19, and less preventive measures against the COVID-19 virus infection, including vaccines.

According to the HBM, feeling less worried about getting COVID-19 post-vaccination and believing it is vaccine-preventable were the most significant predictors of vaccine acceptance, whereas falsely believing HIV medications protect against COVID-19 transmission and having concerns about the vaccines’ effectiveness were stronger predictors for non-acceptance, consistent with similar studies evaluating effectiveness.^35,36^ More than half of the participants were concerned about the vaccine side effects and 47% were concerned regarding potential interactions between their HIV treatment and COVID-19 vaccines.

This result is consistent with the findings of Huang et al.,^25^ which showed that about half of PLHIV respondents in China had concerns related to side effects, and potential interactions between antiretroviral treatment and COVID-19 vaccines. Similarly, Vallée et al.^24^ found that concerns about the serious side effects of COVID-19 vaccines were independently associated with COVID-19 vaccine hesitancy. Similar to studies conducted among the general population in the MENA region, concerns about COVID-19 vaccine safety and fear of side effects were among the most commonly cited reasons for vaccine hesitancy.^20,32,35^

As relatively small numbers of PLHIV have been involved in the phase III COVID-19 vaccine trials, limited data has been available regarding COVID-19 vaccines in this specific population.^37,38,39^ However, the World Health Organization, as well as several international health authorities, has released recommendations for PLHIV to receive COVID-19 vaccines and to address their safety and efficacy concerns.^40,41,42^ These recommendations emphasise no evidence of safety concerns unique to PLHIV and, as with the general population, the vaccines are considered safe. Moreover, PLHIV have been considered as a priority group for the vaccination. Despite being widely reported by participants in our study, there is no compelling evidence to support the use of HIV medications for treatment or prevention of COVID-19, and no evidence on potential interaction between COVID-19 vaccine and HIV drugs.^40,41,42^ These findings are important for PLHIV in this region. Authoritative and trustworthy information sources, such as national AIDS programmes and local health authorities, should engage PLHIV with clear, carefully crafted and consistent messages, communicated in plain, non-stigmatising language and in a way that people of all educational levels can understand, to address their specific concerns and perceived barriers regarding COVID-19 vaccination. Ensuring that PLHIV have adequate access to COVID-19 vaccines, as well as sufficient information to make evidence-based immunisation decisions, should be priorities of national health authorities.

Prior studies among the general population have shown that high perceived susceptibility of getting COVID-19 or perceptions of the severity of COVID-19 were significant predictors for vaccine acceptance.^35,43^ In addition, low perceived severity of COVID-19 infection was highly correlated with COVID-19 vaccine refusal among French PLHIV.^24^ However, among PLHIV in the MENA region, perception of susceptibility and severity of COVID-19 infection were not significant predictors. One explanation for decreased risk perception could be that, because of inadequate testing and underreported incidences of COVID-19 infections and mortalities in countries across the region,^44^ people might consider COVID-19 to be an ordinary seasonal respiratory illness rather than the highly transmissible disease it is. This highlights a need to address risk perception and severity among PLHIV, as high-risk perceptions are important precursors to positive health behaviours, including being a significant predictor of intention to take the COVID-19 vaccine.^45,46^ Low perceived risk may be correlated not only with vaccine acceptance, but also to adherence to social distancing measures and other public health countermeasures, in the absence of vaccine access.^45,46^

Our finding on vaccine acceptance associated with a doctor recommendation, consistent with other studies, demonstrates that healthcare workers, on the frontline of HIV care, can play an influential role in building vaccination literacy and in improving trust in COVID-19 vaccines among PLHIV.^47^ They should be well trained and equipped with the tools to support PLHIV, including listening to their fears and anxieties, answering their questions, and addressing their concerns towards vaccines, as well as correcting misinformation, and being aware of potential stigma concerns.

HIV is a highly stigmatised condition in the MENA region.^48^ The stigmatising and discriminatory attitudes PLHIV face in healthcare settings could deter populations at high risk from seeking health services.^49^ Many civil society organisations and community-based organisations in the region are led by or involve people most affected by HIV and have become integral to the MENA region’s HIV response. Civil society organisations are often more effective in reaching PLHIV and key populations than health authorities^50^ and can therefore play an essential role in building trust in COVID-19 vaccines among these groups. Peer-support for PLHIV can also fulfill a useful role in promoting COVID-19 vaccination, sharing supportive advice and positive experiences of COVID-19 vaccination.

Those reporting more than one year since their last CD4+ cell count check may be less well engaged with the health system and may, in turn, inadvertently accept higher health risks (e.g., remaining unvaccinated) due to lack of information or access to care than those reporting more engagement. Leveraging lessons learned from models of hepatitis C virus/HIV care^51^ may provide practical solutions to make vaccination more convenient for PLHIV without risking disclosure of their HIV status. These include, for example, co-location of vaccination services within HIV clinics, ‘one-stop-shop’ models, and door-to-door administration, and can make vaccines easily accessible in safe, familiar and convenient locations.

Limitations of this study include potential selection bias using web-based survey methods, and the cross-sectional nature, preventing us from drawing any causal conclusions. Although Facebook and WhatsApp platforms were valuable in helping to achieve the minimum sample, selection bias could have been introduced by using these methods of advertisement. Nevertheless, this study is the first to evaluate COVID-19 vaccine acceptance and its associated factors among PLHIV in the MENA region. Such findings should help policymakers planning interventions to improve vaccination coverage among this group.

## Conclusion

This study reports overall COVID-19 vaccine acceptance of 64.6% among PLHIV in three countries of the MENA region. Factors for vaccine acceptance included feeling less worried about COVID-19 transmission post-vaccination, believing COVID-19 is vaccine-preventable, and having a doctor recommendation. Factors against getting vaccinated included living in a rural area, having a most recent CD4+ cell count check more than one year ago, having concerns about vaccine effectiveness and believing HIV medications protect against COVID-19 transmission. Effective strategies are needed to guarantee PLHIV access to COVID-19 vaccines as a priority group for vaccination as well as to increase vaccine acceptance among PLHIV through clearly tailored messages, addressing their specific concerns and improving their knowledge and awareness about the safety and benefits of COVID-19 vaccines.
